# Comparison of cone beam computed tomography measurements obtained after manual and AI-assisted head positioning and reconstruction

**DOI:** 10.1007/s00784-026-07260-5

**Published:** 2026-11-11

**Authors:** Bruno Werlang, Débora Costa Ruiz, Amanda Flor, Monikelly do Carmo Chagas Nascimento, Anne Caroline Oenning

**Affiliations:** 1 Division of Oral Radiology, Faculdade São Leopoldo Mandic, Instituto de Pesquisas São Leopoldo Mandic, Campinas, SP Brazil; 2 Department of Clinical Dentistry, Federal University of Espírito Santo (UFES), Espírito Santo, Brazil; 3 Department of Oral Diagnosis, Piracicaba Dental School, University of Campinas, Piracicaba, SP Brazil

**Keywords:** Cone beam computed tomography, Dental implants, Diagnosis, Radiology

## Abstract

**Objectives:**

To compare measurements obtained from cone beam computed tomography (CBCT) exams by radiologists following manual head positioning and panoramic curve tracing with those obtained after artificial intelligence (AI)-assisted positioning adjustments performed prior to measurement.

**Materials and methods:**

Twenty-five CBCT exams presenting edentulous sites corresponding to missing mandibular first molars were selected. Five examiners performed measurements of bone height and width after manual head positioning adjustments and panoramic curve tracing (manual method). Subsequently, the same exams were processed using TomoNav software, which automatically performed volume alignment and generated the panoramic curve and cross-sectional images. The examiners then repeated the measurements (AI-assisted method). Measurements obtained from both approaches were summarized using descriptive statistics and compared using the intraclass correlation coefficient (ICC), paired t-test, and Bland–Altman analysis (p˂0.05).

**Results:**

Excellent inter-examiner reproducibility was observed for both height and width measurements, with ICC values of 0.94 for height and 0.81 and 0.85 for width using the manual and AI-assisted methods, respectively. Significant differences were identified for left-side height, total height, right-side width, and total width (*p* < 0.05). However, the mean biases were low and overall agreement between methods was excellent, with ICC values equal or greater than 0.89.

**Conclusion:**

Measurements obtained with the AI-assisted method for volume repositioning and generation of panoramic and cross-sectional images were comparable to those obtained using a conventional method.

**Clinical relevance:**

AI facilitates CBCT implant-planning preprocessing without compromising measurement reproducibility.

## Introduction

Cone-beam computed tomography (CBCT) has become an essential imaging modality for dental implant planning because it provides accurate three-dimensional assessment of the area of interest, allowing the identification of critical anatomical structures, as well as the evaluation of bone quality and availability [1–5]. Due to its high dimensional precision, CBCT contributes to more reliable measurements of potential surgical sites, making treatment safer and more predictable [6].

After CBCT acquisition, multiplanar reconstructions are generated, and a panoramic curve is traced along the alveolar ridge. Based on this curve, cross-sectional images are reconstructed and used for linear bone measurements and sequential buccolingual evaluation of edentulous sites during preoperative implant planning [7]. A critical step in obtaining these sections is prior alignment of the dental arch according to reference planes, correcting head inclinations that occurred during image acquisition and promoting more reproducible measurements among operators [8]. A recent study investigated whether different methods of panoramic curve tracing could influence implant planning [9]. The authors observed that distinct manual tracing methods did not significantly affect clinical outcomes [9]. In addition to manual methods, studies proposing semiautomatic and automatic techniques for dental arch definition and panoramic reconstruction from CBCT exams have shown promising results and the potential to reduce manual intervention [10, 11].

In this context, artificial intelligence (AI) has been increasingly integrated into dentomaxillofacial radiology and dental implantology [12]. A previous study described an AI-based automated method for panoramic reconstruction that employs a 3D U-Net model to adapt the dental arch surface and generate a three-dimensional reconstruction zone centered on the teeth [13]. This approach produced panoramic radiographs with improved sharpness, better alignment, and enhanced contrast across the entire dental arch [13].

However, evidence regarding the reliability of measurements obtained by these tools for implant planning remains limited. This gap is clinically relevant, since automated systems have the potential to reduce operator-dependent steps in the workflow and optimize professional workload [14]. Therefore, the aim of the present study was to compare measurements obtained from CBCT exams by radiologists following manual head positioning and panoramic curve tracing with those obtained after AI-assisted positioning adjustments performed prior to measurement.

## Materials and methods

The present research was conducted after approval from the local Institutional Research Ethics Committee (protocol number CAAE 88251425.0.0000.5374), without any restrictions, and in accordance with the Helsinki Declaration.

### Sample selection and preparation

Based on a previous study [9], a sample size of twenty-five mandibular CBCT exams belonging to patients that needed implant planning were selected from the image database of the Oral Radiology Clinic at São Leopoldo Mandic School of Dentistry (Campinas, SP, Brazil). The scans were obtained using an OP300 Maxio (Instrumentarium, Tuusula, Finland) with the following parameters: 8 × 6 cm field of view, 0.2-mm voxel size, 90 kVp, and 8–11 mA. Eligible CBCT scans were required to present a single edentulous site corresponding to the absence of the mandibular first molar, as this region was selected to standardize the measurement site across all examinations. Scans presenting pathological alterations adjacent to the region of interest or exhibiting imaging artifacts, such as hypodense or hyperdense streaks caused by high–atomic number materials, were excluded.

After sample selection, all CBCT exams were exported in Digital Imaging and Communications in Medicine format and anonymized by one of the authors of the present study (B.W.) to avoid potential bias during image assessment.

### Image evaluation (manual method)

**Fig. 1:**
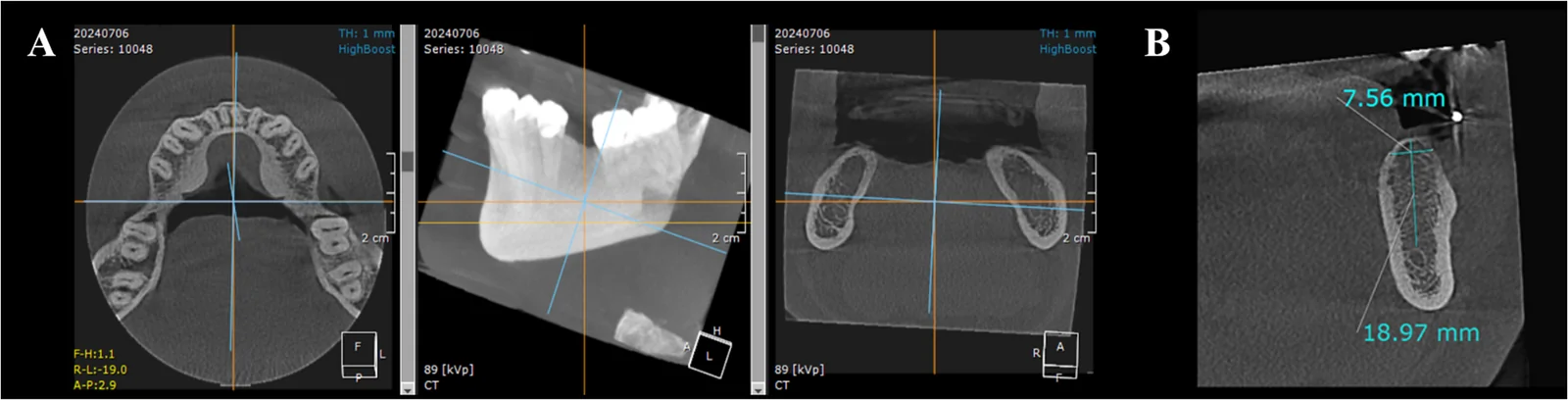
Representative images of the manual method: Head positioning before panoramic curve tracing (**A**) followed by measurements of bone height and width, both performed by operators (**B**)

The 25 CBCT scans were independently evaluated by five oral and maxillofacial radiologists with at least five years of experience in three-dimensional image interpretation. Initially, each examiner assessed the scans using OnDemand 3D software (Cybermed Inc, Seoul, Korea). They manually reoriented each CBCT volume using the multiplanar reconstruction views. The axial, sagittal, and coronal planes were adjusted according to the individual mandibular anatomy, adopting an intermediate reference between the occlusal and mandibular planes. Following reorientation, the panoramic curve was manually positioned along the mandibular arch and adapted to the morphology of the edentulous region to generate the corresponding cross-sectional images (Fig. 1A). On these cross-sectional images, mandibular bone height and width were measured at the center of the edentulous site corresponding to the mandibular first molar region. Bone height was defined as the distance from the alveolar crest to the superior cortical border of the mandibular canal. Bone width was defined as the distance between the buccal and lingual cortical plates, measured approximately 2 mm below the alveolar crest (Fig. 1B).

Before the assessment, the examiners were calibrated using 10 CBCT examinations that were not included in the final sample, allowing them to clarify any questions regarding the evaluation procedure.

### Image evaluation (AI-assisted method)

**Fig. 2:**
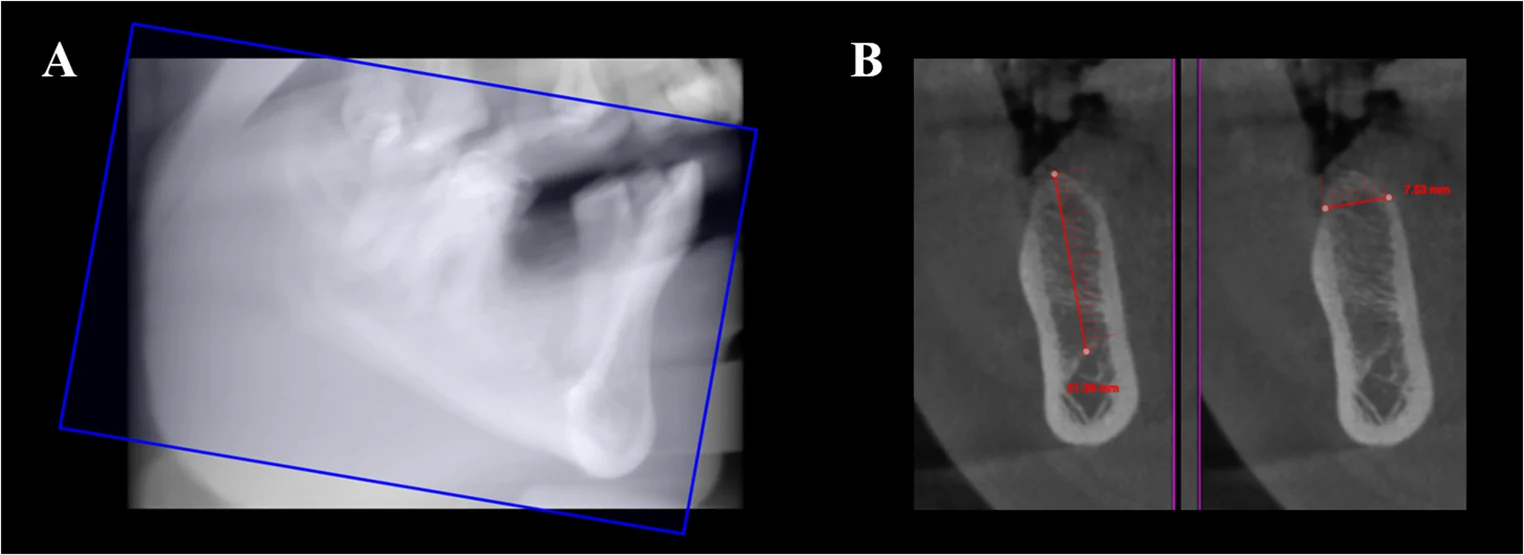
Representative images of the AI-assisted method: Head positioning before panoramic curve tracing performed by the AI-tool (**A**) followed by measurements of bone height and width performed by the operators (**B**)

After a 10-day interval, the CBCT examinations were re-anonymized and independently re-evaluated by the same examiners using the TomoNav platform (Radio Memory, Belo Horizonte, Brazil), which employs a proprietary AI algorithm. In this second evaluation, the software automatically performed the three-dimensional volume orientation (Fig. 2A) and generated the panoramic curve and corresponding cross-sectional images, without any manual intervention. Subsequently, the same five oral radiologists performed the linear measurements using only the reconstructions generated by the software, while maintaining the same anatomical reference points previously established for the manual method (Fig. 2B).

Both assessments occurred in a quiet and low-light environment. Aiming to prevent visual fatigue, the examiners were instructed to analyze no more than 10 CBCT exams per day. To replicate a more clinical scenario, adjustments on brightness and contrast and the use of the zoom tool were allowed.

### Statistical analysis

Statistical analyses were conducted using R software (R Foundation for Statistical Computing, Vienna, Austria), with the significance level set at 5% (*p* < 0.05). For each method (manual and AI-assisted), mean values, standard deviations (SD), and quartiles were calculated based on the measurements obtained by the five examiners. Descriptive statistics were used to assess variability among examiners, and inter-examiner agreement was evaluated using two-way random-effects intraclass correlation coefficient (ICC), with absolute agreement and average measurements, calculated separately for each variable and method.

Subsequently, for each variable (bone height and bone width), the mean of the measurements obtained by the five examiners was calculated and used for comparisons between methods. Differences between the mean values were assessed using the paired t-test. Reproducibility between methods was evaluated using a two-way mixed-effects intraclass correlation coefficient with absolute agreement and single measurements, based on the examiner-averaged values for each method, and the Bland–Altman method. In the Bland–Altman analysis, the bias between the measurements obtained with the two methods was calculated, together with the corresponding 95% limits of agreement.

## Results

Table 1 presents the descriptive analysis of height measurements obtained by each examiner. Using the manual method, mean values ranged from 13.26 mm to 15.02 mm on the right side and from 12.52 mm to 14.78 mm on the left side. With the AI-assisted method, mean height values ranged from 13.22 mm to 15.05 mm on the right side and from 13.05 mm to 15.05 mm on the left side. Table 2 shows the width measurements. Using the manual method, mean values ranged from 4.31 mm to 7.19 mm on the right side and from 4.44 mm to 6.81 mm on the left side. With the AI-assisted method, width measurements ranged from 4.26 mm to 6.26 mm, and from 4.09 mm to 6.42 mm on the right and left sides, respectively.

**Table 1:** Mean (SD) and median (IQR) of height measurements obtained by five examiners (mm)

Examiners	Metrics	Manual method		AI–assisted method	
		Right	Left	Right	Left
1	Mean	13.90 (2.63)	14.00 (2.85)	14.63 (2.64)	15.05 (2.57)
	Median	14.60 (13.21— 15.61)	14.53 (12.50— 16.21)	14.45 (12.93— 16.42)	15.05 (12.75— 17.27)
2	Mean	14.70 (2.29)	14.35 (2.75)	15.05 (2.73)	14.71 (2.90)
	Median	14.87 (12.41— 15.90)	14.17 (12.52— 15.99)	15.06 (13.04— 16.82)	14.70 (12.66— 16.50)
3	Mean	13.26 (2.43)	12.52 (3.02)	13.22 (2.15)	13.43 (2.72)
	Median	12.94 (11.95— 14.96)	12.34 (11.26— 14.14)	12.97 (12.13— 14.55)	13.40 (11.47— 15.59)
4	Mean	13.93 (2.41)	13.70 (2.94)	13.45 (2.59)	13.05 (2.75)
	Median	13.72 (12.41— 15.87)	13.85 (11.31— 16.35)	13.05 (11.17— 15.05)	13.41 (11.35— 14.55)
5	Mean	15.02 (2.25)	14.78 (2.31)	15.05 (2.56)	14.89 (3.24)
	Median	15.28 (13.76— 16.72)	15.36 (12.74— 16.64)	15.08 (13.12— 16.96)	15.06 (12.74— 17.51)

*SD* standard deviation, *IQR* interquartile range

**Table 2:** Mean (SD) and median (IQR) of width measurements obtained by five examiners (mm)

Examiners	Metrics	Manual method		AI–assisted method	
		Right	Left	Right	Left
1	Mean	5.97 (1.75)	5.44 (1.59)	5.56 (1.87)	5.50 (1.67)
	Median	5.80 (4.55— 6.92)	5.03 (4.41— 5.70)	5.32 (4.02— 6.36)	5.19 (4.62— 5.97)
2	Mean	7.19 (1.98)	6.81 (1.30)	6.26 (1.56)	6.20 (2.09)
	Median	7.28 (5.54— 8.62)	6.53 (5.93— 7.38)	6.07 (4.91— 7.31)	5.76 (4.97— 6.55)
3	Mean	4.31 (0.92)	4.55 (1.63)	4.26 (1.10)	4.31 (1.29)
	Median	4.28 (3.64— 4.54)	3.90 (3.63— 4.6)	3.99 (3.59— 4.53)	4.04 (3.55— 4.49)
4	Mean	4.42 (1.19)	4.44 (1.39)	4.41 (1.06)	4.09 (0.66)
	Median	4.19 (3.65— 4.43)	4.04 (3.61— 4.32)	4.12 (3.70— 4.54)	3.89 (3.56— 4.49)
5	Mean	6.22 (1.68)	5.99 (1.51)	6.24 (1.90)	6.42 (2.24)
	Median	6.12 (4.78— 7.08)	5.59 (5.13— 6.17)	6.08 (4.51— 7.19)	5.91 (5.24— 6.28)

*SD* standard deviation, *IQR* interquartile range

When height measurements were performed using the manual method, the mean differences between examiners ranged from 0.13 mm to 2.01 mm. However, when using the AI-assisted method, mean differences ranged from 0.04 mm to 1.72 mm. Regarding width measurements, differences between examiners ranged from 0.01 mm to 2.57 mm with the manual method, and from 0.03 mm to 2.08 mm with the AI-assisted method, as seen in Table 3.

**Table 3:** Differences among examiners in measurements (mm) obtained by the two methods, expressed as mean (SD) and median (IQR) *SD* Standard Deviation, *IQR* interquartile range

Examiners	Manual method				AI–assisted method			
	Height		Width		Height		Width	
	Mean	Median	Mean	Median	Mean	Median	Mean	Median
1–2	−0.57 (1.46)	−0.35 (−1.10— 0.12)	−1.29 (0.96)	−1.32 (−1.63— −0.71)	−0.04 (1.75)	−0.21 (−0.72— 0.20)	−0.70 (0.89)	−0.62 (−1.13— −0.27)
1–3	1.06 (2.04)	0.87 (0.45— 1.65)	1.27 (1.50)	1.25 (0.68— 2.24)	1.52 (1.17)	1.36 (0.89— 1.86)	1.24 (0.86)	1.23 (0.43— 1.74)
1–4	0.13 (1.49)	−0.03 (−0.54— 1.18)	1.28 (1.30)	1.03 (0.77— 1.97)	1.59 (1.62)	1.29 (0.50— 2.24)	1.28 (1.22)	1.20 (0.33— 1.80)
1–5	−0.95 (1.47)	−0.62 (−1.59— −0.17)	−0.40 (0.92)	−0.35 (−0.83— 0.18)	−0.13 (1.41)	−0.23 (−0.76— 0.25)	−0.80 (1.16)	−0.52 (−1.22— −0.18)
2–3	1.63 (1.56)	1.42 (0.62— 2.14)	2.57 (1.45)	2.42 (1.82— 3.44)	1.55 (1.26)	1.58 (1.02— 2.08)	1.95 (0.95)	1.88 (1.35— 2.52)
2–4	0.70 (1.50)	0.79 (−0.08— 1.63)	2.57 (1.12)	2.50 (1.76— 3.33)	1.63 (1.32)	1.53 (0.81— 2.10)	1.98 (1.28)	1.67 (1.14— 2.31)
2–5	−0.38 (1.20)	−0.20 (−0.94— 0.30)	0.90 (0.71)	0.88 (0.41— 1.34)	−0.09 (2.03)	−0.03 (−0.30— 0.29)	−0.10 (1.11)	−0.02 (−0.35— 0.41)
3–4	−0.93 (1.75)	−0.75 (−1.53— 0.10)	0.01 (0.94)	0.01 (−0.23— 0.26)	0.08 (1.35)	−0.18 (−0.79— 0.74)	0.03 (0.74)	−0.08 (−0.30— 0.17)
3–5	−2.01 (1.54)	−1.84 (−2.47— −1.18)	−1.67 (1.34)	−1.57 (−2.25— −1.06)	−1.65 (1.66)	−1.64 (−2.31— −1.02)	−2.05 (1.35)	−2.01 (−2.62— −1.25)
4–5	−1.09 (1.16)	−0.97 (−1.43— −0.45)	−1.68 (0.85)	−1.69 (−2.32— −1.08)	−1.72 (1.99)	−1.52 (−2.05— −1.09)	−2.08 (1.54)	−1.89 (−2.69— −1.13)

*SD* Standard Deviation, *IQR* interquartile range

As presented in Table 4, inter-examiner reproducibility was excellent for height measurements, with an ICC of 0.94 (95% CI: 0.89–0.97) for the manual method and 0.94 (95% CI: 0.87–0.97) for the AI-assisted method. For width measurements, reproducibility was also excellent with both methods, with ICC values of 0.81 (95% CI: 0.54–0.91) for the manual method and 0.85 (95% CI: 0.63–0.96) for the AI-assisted method.

**Table 4:** Intraclass correlation coefficients among examiners for the measurements obtained by the two methods tested

METHOD	MEASUREMENT	ICC (95% CI)
MANUAL	Height	0.94 (0.89–0.97)
	Width	0.81 (0.54–0.91)
AI–ASSISTED	Height	0.94 (0.87–0.97)
	Width	0.85 (0.63–0.96)

Considering the mean values of the five examiners, a statistically significant difference was observed between the two methods for left-side height and for height regardless of side, as well as for right-side width and for width regardless of side (*p* < 0.05), as observed in Table 5. The mean biases between the two methods, calculated using the Bland–Altman approach, ranged from 0.09 mm (left-side width) to 0.61 mm (left-side height). For height and width regardless of side, the mean biases between methods were 0.44 mm and 0.22 mm, respectively. The ICC values indicated excellent agreement between the two methods. As presented in Table 6, the AI-assisted method yielded higher height measurements in 74% of cases, whereas the manual method yielded higher width measurements in 74% of cases.

**Table 5:** Reproducibility of measurements (mm) by method, summarized across the five examiners using mean (SD) and median values

Measurement	Method				^1^*p*-value	^2^Bias (95% LoA)	ICC (95% CI)
	Manual		AI – assisted method				
	Mean (SD)	Median ´(minimum–maximum)	Mean (SD)	Median (minimum-maximum)			
Right-side height	14.22 (2.29)	14.33 (10.02–19.22)	14.49 (2.36)	14.59 (9.54 − 20.84)	0.1280	−0.27 (−1.94–1.40)	0.96 (0.92–0.98)
Left-side height	13.91 (2.50)	13.99 (8.63–17.71)	14.52 (2.69)	14.92 (9.24 − 19.49)	0.0014	−0.61 (−2.25–1.04)	0.96 (0.87–0.99)
Height*	14.07 (2.38)	14.16 (8.63–19.22)	14.50 (2.50)	14.75 (9.24 − 20.84)	0.0007	−0.44 (−2.11–1.24)	0.96 (0.91–0.98)
Right-side width	5.92 (1.50)	5.86 (4.00–10.12)	5.58 (1.55)	5.72 (3.59–9.74)	< 0.0001	0.34 (−0.44–1.13)	0.97 (0.83–0.99)
Left-side width	5.70 (1.29)	5.36 (4.15–8.71)	5.61 (1.69)	5.23 (3.73–11.02)	0.6501	0.09 (−1.79–1.96)	0.89 (0.75–0.95)
Width*	5.81 (1.39)	5.37 (4.00–10.12)	5.59 (1.61)	5.29 (3.59–11.02)	0.0432	0.22 (−1.23–1.66)	0.93 (0.88–0.96)

^1^Measurements were analyzed with paired T-Test (significance level of 5%). ^2^Mean bias between the two methods, with 95% limits of agreement, calculated using the Bland-Altman method. *LoA* limits of agreement, *ICC* Intraclass Correlation Coefficient with 95% confidence interval

^*^Irrespective of side

**Table 6:** Frequency distribution of measurements with higher values according to the assessed method

Measurement	Manual > AI-assisted	AI- assisted > Manual
	Frequency (%)	
Right-side height	9 (36.0%)	16 (64.0%)
Left-side height	4 (16.0%)	21 (84.0%)
Height* 8	13 (26.0%)	37 (74.0%)
Right-side width	20 (80.0%)	5 (20.0%)
Left-side width	17 (68.0%)	8 (32.0%)
Width*	37 (74.0%)	13 (26.0%)

*Irrespective of side

## Discussion

Considering the critical importance of accurate implant treatment planning, as errors may lead to implant misfit, surgical complications, or even implant removal, and given the increasing integration of AI tools into treatment planning workflows [2, 3], the present study evaluated whether linear measurements obtained from CBCT exams after automated AI-based processing are comparable to those obtained through the conventional workflow performed exclusively by human operators. Overall, the findings demonstrated high agreement between methods, suggesting that automated generation of volume alignment and panoramic curves did not compromise the bone height and width measurements.

The highest inter-examiner reproducibility values for both methods were found for height measurements (0.94 for manual and AI-assisted methods), indicating that vertical measurements were highly consistent. This result is expected, once height measurements in the posterior mandible are generally based on more stable and anatomically well-defined landmarks compared to the maxillary bone, which may reduce operator-dependent variability. From a clinical perspective, this is relevant because vertical bone height is a critical parameter in implant planning, especially in relation to the mandibular canal and the selection of implant length [15].

Regarding width measurements, the manual and AI-assisted methods yielded ICC of 0.81 and 0.85, respectively. Although these ranges are similar, the slightly higher ICC observed with the AI-assisted method suggests that automated panoramic curve tracing and cross-sectional slice generation may help limit some of the operator-dependent variability associated with manual reconstruction. Therefore, these preprocessing steps may also warrant careful consideration when measuring the area of interest. Bone width measurements might be more sensitive to subtle differences in slice angulation and positioning of the cross-sectional image, and even minor variations in reconstruction geometry can affect the measured buccolingual dimension. In this scenario, the improved consistency observed with the AI-assisted method reflects the benefit of a more standardized reconstruction process.

The aforementioned finding also helps explain another result of the study. A consistent pattern was observed in which height measurements were greater with the AI-assisted method, whereas width measurements were greater with the manual method in approximately 74% of cases. A plausible explanation lies in inherent differences in the reconstruction workflows. Because alveolar ridge dimensions are sensitive to reconstruction parameters, even subtle variations in preprocessing steps may influence height and width [16]. This may lead to systematically greater height values in the AI-assisted workflow and greater width values in the manual workflow.

Although the AI-assisted method incorporated an AI-based approach, the linear bone measurements were still performed by human operators: the AI tool contributed to implant planning by automating image preprocessing steps, while the final measurements remained dependent on human interpretation. This interaction illustrates how AI can be effectively integrated into the diagnostic workflow as a supportive tool, enhancing efficiency without replacing clinical judgment [17]. Maintaining clinician involvement in the decision-making process is essential, as it enables the identification and correction of potential inconsistencies arising from automated processing.

By contrast, fully automated AI-based workflows have already been described in the literature, in which the entire planning process is performed with no human intervention [18]. Such workflows have been explored in orthodontics for clear aligner treatment planning. A previous scoping review demonstrated that current digital protocols already include a sequence of automated steps, such as segmentation of anatomical structures from CBCT exams, fusion of CBCT datasets with intraoral scans, digital planning of tooth movement, definition of attachment positioning, and fabrication of clear aligner models. This scenario appears to differ from implant dentistry [18]. Elgarba et al. [19] evaluated the application of AI in digital implant planning and concluded that a fully automated workflow has not yet been documented or scientifically validated [19]. However, even without a fully automated workflow, the use of AI in specific implant planning steps may still provide clinical benefits, once these tools can improve time efficiency by reducing operator workload [19].

Both aforementioned scoping reviews reported that studies evaluating AI-based tools frequently lacked detailed descriptions of the underlying algorithms [18, 19]. This is partially attributable to the proprietary nature of many commercially available AI systems. The same limitation applies to the present study, as the manufacturer of the TomoNav platform, which was used to automatically generate the panoramic curve, does not publicly disclose technical information regarding the underlying AI architecture, training dataset, or implementation (e.g., landmark detection, volume registration, or deep learning model) [20]. Consequently, these details were not available for further discussion. The limited disclosure of technical information primarily reflects manufacturers’ efforts to protect intellectual property and preserve their competitive advantage. Furthermore, because these AI systems are continuously refined through software updates, manufacturers may choose not to publicly disclose technical specifications that could become outdated following subsequent revisions.

The CBCT examinations included in this study were acquired at a single institution; however, this is unlikely to have substantially influenced the results obtained with the AI-assisted method. The software is designed for use with CBCT examinations obtained in routine clinical practice, irrespective of the CBCT device [20]. Therefore, the present findings are likely to reflect the software’s performance under comparable clinical conditions. Nevertheless, multicenter studies including examinations from different institutions and CBCT units would be valuable to further validate the generalizability of the results. Another limitation is that different software platforms had to be used for the manual and AI-assisted methods, as OnDemand 3D does not support automated panoramic curve generation, whereas TomoNav does not offer a manual curve-generation option. Importantly, the comparison was intended to evaluate the clinical workflow provided by the AI-assisted approach against the conventional manual workflow, rather than to isolate the effect of panoramic curve generation from the software platform itself. Future studies should also evaluate other AI-assisted platforms. Previous research found that the AI-based radiological reporting tool in the Diagnocat software (Diagnocat Inc., San Francisco, CA, USA) performed satisfactorily in identifying teeth, determining the number of roots and canals, assessing crown condition, and detecting endodontic treatment on CBCT scans [21]. However, many AI tools used in clinical practice, including those capable of generating panoramic curves, have yet to be independently validated; therefore, further studies are needed to determine whether the present findings can be generalized across different software platforms.

Although statistically significant differences were observed for left-side height and total height, as well as right-side width and total width, their magnitude was small in most cases. This interpretation was further supported by the Bland–Altman analysis, which demonstrated low overall mean biases between the methods. These findings emphasize the importance of distinguishing statistical significance from clinical relevance, as the observed differences in linear measurements are unlikely to influence most clinical situations. This highlights that human landmark selection remains a primary source of variability in imaging planning. In this context, AI-assisted workflows hold strong potential to enhance measurement consistency by standardizing volume orientation and providing automated reference points. Moreover, for bone height, discrepancies of less than 2 mm are generally not expected to affect implant selection or positioning because a safety margin is routinely maintained between the implant apex and critical anatomical structures, such as the mandibular canal, to minimize the risk of complications, including inferior alveolar nerve injury, hemorrhage, and neurosensory disturbances [22–26].

The study sample consisted of CBCT examinations originally acquired for clinical purposes unrelated to this research. Consequently, no gold standard reference was available, as establishing one would require direct measurements on the corresponding dry mandibles [27]. However, this should be interpreted within the clinical context of the study. Unlike in vitro investigations, the present study analyzed real patient CBCT scans, preserving the presence of soft tissues and the natural anatomical variability encountered in clinical practice rather than relying on simulated models using materials such as water or wax [28]. Additionally, the accuracy of CBCT-derived linear measurements for implant planning has been extensively validated in the literature, including under low-dose acquisition protocols [5, 29–31].

Intra-examiner agreement was not assessed in the present study based on findings reported by Adames et al. [9]. In that study, 20 clinicians performed panoramic curve tracing before generating cross-sectional reconstructions for implant planning. Although variations in curve tracing resulted in small differences in the measurements, they did not significantly affect the outcomes, and intra-examiner agreement ranged from good to excellent across all cases [9]. Therefore, the absence of intra-examiner analysis in the present study was considered unlikely to compromise our results.

In all cases, measurements were performed for implant planning in the mandibular first molar region. The measurement slice was not predefined according to a specific anatomical or numerical criterion, allowing examiners to select the slice as they would in routine clinical practice. This approach was intended to reproduce the operator-dependent variability inherent to implant planning. The mandibular first molar region was intentionally selected based on tooth type to standardize the sample, as the mandibular first molar is the first permanent tooth to erupt and, consequently, one of the teeth most frequently lost, making it a common site for implant-supported rehabilitation [32]. Future studies should investigate whether similar results are obtained in different edentulous conditions, particularly in the maxillary arch and in larger edentulous spaces involving the loss of multiple teeth rather than the single-tooth edentulous site evaluated in the present study.

Moreover, since previous studies have shown that both the application of image processing adjustments and the specific CBCT unit used can influence the visualization of critical anatomical structures for implant planning, such as the mandibular canal, further research should investigate cases involving scans with imaging artifacts, as well as scans acquired using different CBCT devices, especially those equipped with image enhancement resources such as metal artifact reduction algorithms [33–35]. These investigations may provide a broader understanding of how manual and AI-assisted measurement methods perform under more challenging and clinically representative conditions.

Finally, the findings of the present study suggest that AI-assisted reconstruction may offer potential clinical benefits by automating an operator-dependent step of the implant planning workflow. This approach may improve workflow efficiency. Although this potential benefit was not directly evaluated in the present study, future studies should investigate the effects of AI-assisted reconstruction on processing time, operator dependency, and performance across different levels of clinical experience to further clarify its role in routine implant planning.

## Conclusion

Based on the present findings, CBCT measurements obtained using the AI-assisted TomoNav software for volume repositioning and cross-sectional image generation were comparable to those obtained using the conventional method performed exclusively by human operators. Further evaluation of other software platforms is warranted to determine the generalizability of these findings.

## Data Availability

No datasets were generated or analysed during the current study.
